# PERSIAN Eye Cohort Study (PECS): Design, Methodology

**DOI:** 10.34172/aim.2023.70

**Published:** 2023-08-01

**Authors:** Fateme Alipour, Hassan Hashemi, Alireza Lashay, Fatemeh Jafari, Nazgol Motamed-Gorji, Mahmoud Jabbarvand Behrouz, Mohammad Mirzaei, Yousef Alizade, Mohammad Reza Soleymani, Mohammad Reza Shoja, Kourosh Shahraki, Gholam Reza Khataminia, Hossein Poustchi, Reza Malekzadeh

**Affiliations:** ^1^Translational Ophthalmology Research Center, Farabi Eye Hospital, Department of Ophthalmology, Tehran University of Medical Sciences, Tehran, Iran; ^2^Noor Ophthalmology Research Center, Tehran, Iran; ^3^Digestive Disease Research Institute, Shariati Hospital, Tehran University of Medical Sciences, Tehran, Iran; ^4^Department of Ophthalmology, Tabriz University of Medical Sciences, Tabriz, Iran; ^5^Amiralmomenin hospital, Department of ophthalmology, Gillan University of Medical Sciences, Rasht, Iran; ^6^Department of Ophthalmology, Rafsanjan University of Medical Sciences, Rafsanjan, Iran; ^7^Department of Ophthalmology, Shahid Sadoughi University of Medical Sciences, Yazd, Iran; ^8^Department of Ophthalmology, Zahedan University of Medical Sciences, Zahedan, Iran; ^9^Department of Ophthalmology, Jundishapur University of Medical Sciences, Ahvaz, Iran; ^10^Liver, Pancreatic, and Biliary Diseases Research Center, Digestive Diseases Research Institute, Tehran University of Medical Sciences, Tehran, Iran

**Keywords:** Cohort study, Epidemiology, Iran, Ophthalmology

## Abstract

**Background:**

To report the study protocol, methodology and latest enrollment data of a large epidemiological multi-central eye cohort named PERSIAN Eye Cohort Study (PECS), originating from the ongoing PERSIAN Cohort Study, to investigate the distribution of ophthalmic disorders in different regions and ethnicities of Iran, and determine their associations with various exposures of ophthalmic and non-ophthalmic nature.

**Methods:**

A central committee designed the study and equipped six chosen centers (Khameneh, Some’e Sara, Hoveizeh, Yazd, Rafsanjan and Zahedan). A focal point in each center conducted the study under close supervision of the central committee.

**Results:**

This ongoing study was launched in 2014. Out of 65,580 eligible participants of the PERSIAN Cohort, 48,618 individuals aged 35-70 have been enrolled in the PECS (response rate: 74.13%) until June 2021. Slit lamp and fundus photography were performed for 28,702 (59.03%) and 27,437 (56.43%) individuals, respectively.

**Conclusion:**

This large epidemiological multi-central eye cohort can improve our epidemiological knowledge of the prevalent ophthalmic disorders in different regions and ethnicities of Iran, and determine their associations with various exposures of ophthalmic and non-ophthalmic nature. This will be very useful for future planned nationwide and global interventions.

## Introduction

 On the global level, population aging has resulted in higher proportions of elderly and middle-aged populations during the last decades. Consequently, age-related chronic disorders are now more prevalent compared to the past, with eye diseases being no exception. Age-related chronic eye diseases such as cataract, age-related macular degeneration (ARMD) and glaucoma are now the main causes of blindness and severe visual impairment (VI) among adults aged 50 years and older around the world.^1^ A meta-analysis in 2016 estimated a 64% increase in the global prevalence of visual impairment secondary to diabetic retinopathy from 1990 to 2010, which was even more prominent in developing regions such as Sub-Saharan Africa, Middle East and North Africa (MENA) and South Asia.^2^

 Many systemic diseases such as diabetes, atherosclerosis and hypertension have been shown to be associated with the development or progression of numerous ophthalmic diseases.^3^ On the other hand, more and more evidence today suggests a role for ophthalmic diseases in predicting future affliction with systemic diseases, such as strokes,^4^ heart failure,^5^ or even mortality.^6^ Therefore, in order to be able to prevent both eye and systemic diseases, clarifying such associations would be beneficial for every population. What strengthens the significance of studying these associations is the fact that many age-related visual impairments as well as their risk factors are preventable. In a recent study by the Global Burden of Disease (GBD), the majority of cases of blindness and moderate-to-severe visual impairment in individuals aged 50 years and older between the years 1990 and 2020 were attributed to cataract and uncorrected refractive errors, two eye conditions with highly preventable vision loss.^1^ The variations in trends, prevalence and patterns of different risk factors and diseases across different geographical regions, or in one region over time, underscores the importance of investigating the epidemiology of ophthalmic diseases in relation to systemic diseases in each distinct population.

 In comparison to high-income countries, low- and middle-income countries are estimated to have higher prevalence of visual impairment. The MENA region (or East-Mediterranean Region, EMR) is one of the areas with a high prevalence of visual impairment and blindness among individuals above 50 years.^7^ Iran is one of these countries.

 Located in the MENA region, Iran is considered an upper-middle-income country, with a population of more than 80 million individuals.^8,9^ A few population-based studies have investigated the epidemiology of eye diseases in Iran, including the Tehran Eye study,^10^ Shahroud Eye cohort^11^ and Zahedan Eye Study^12^; however, information on systemic diseases are limited in these studies. Furthermore, since all of them were conducted with a single-center design, they do not represent different Iranian ethnicities. The Zahedan and Tehran Eye Studies had cross-sectional designs.^10-12^

 The Iranian people consist of several different ethnicities, including Persian, Turk (Azari), Kurd, Lur, Balouch, Arab, etc, scattered geographically across Iran. Furthermore, Iran has different climate zones in different areas, including hot and cold desert, semi-arid steppe, hot Mediterranean and hot summer continental.^13^ Different ethnicities and climates cause different cultural habits and lifestyles, which could result in various exposures among the people of Iran. This could be reflected in different patterns of systemic and ophthalmic diseases throughout Iran. Therefore, when studying the association of eye diseases and systemic diseases in Iran, observing the major ethnic groups as well as different geographical regions is of utmost significance.

 In order to be able to determine the characteristics of ophthalmic and non-ophthalmic disorders in a specific country such as Iran, we need a study with a design as follows:

(1) It should be prospective, meaning that it should collect the data for all relevant exposures, follow the participants over time, and look for the outcomes, including eye diseases. We could be more confident of the causality among different variables in a prospective study design.
(2) It should encompass data on both ophthalmic and non-ophthalmic (systemic) diseases and risk factors.
(3) The study sample should be a comprehensive representation of all major ethnic groups and different climates people live in.

Therefore, according to these criteria, a prospective cohort study targeting all major diseases, including ophthalmic and systemic would be required.

 Launched in 2014, the Prospective Epidemiological Research Studies in IrAN (PERSIAN) (http://persiancohort.com) is a nationwide multisite cohort study to promote research in the field of epidemiology. This cohort includes participants from different regions of Iran, and it has collected a wide array of information with regard to the medical, nutritional and lifestyle situation of its participants, in addition to establishing a large biobank for storing vast amounts of biological samples from all participants. The baseline phase of the PERSIAN cohort was completed in June 2020.^14^

 Since the PERSIAN Cohort’s design fulfills the three aforementioned criteria (in it being prospective, containing the data of systemic diseases, and targeting a wide range of participants from different parts of Iran and different ethnicities), the steering committee decided to initiate an ophthalmic branch of the PERSIAN Cohort in some already-established cohort sites, entitled the PERSIAN Eye Cohort Study (PECS). Accumulating the information of eye health with the data of the main cohort, the PECS aims to:

1. determine the distribution of visual impairment and blindness in different regions of Iran and evaluate the underlying causes,
2. determine the distribution of refractive errors in different regions of Iran,
3. determine the distribution of different eye disorders in different regions of Iran, including diabetic retinopathy, cataract and glaucoma,
4. determine the association between different ophthalmic and non-ophthalmic (systemic) risk factors and ophthalmic disorders in Iran,
5. determine the association of specific Iranian genetic factors and ophthalmic disorders using the PERSIAN Cohort Biobank,
6. aid in improving the quality of eye care and ophthalmic health in different regions of Iran by evaluating the burden of ophthalmic disorders.

The PECS would provide a prospect to use evidence-based recommendations for future eye health care policies in Iran. This report aims to describe the main features of the PECS, methodology and basic findings to date.

## The Main PERSIAN Cohort Study

 The PERSIAN Cohort Study is a prospective study including 165 660 Iranian residents aged between 35 and 70 years, selected from 18 geographically distinct regions of Iran. The design of the PERSIAN Cohort Study was approved by the ethics committees of the Ministry of Health and Medical Education (MOHME), Tehran University of Medical Sciences and relevant participating universities of medical sciences. The MOHME was the major funder for the whole study, while each university was responsible for part of its funding and infrastructure. Written informed consent was obtained from all cohort participants at enrollment.^14,15^

 The baseline phase of the PERSIAN Cohort was launched in 2014 and was completed in 2020. In this phase, comprehensive web-based questionnaires and physical examinations were administered, and biological samples were obtained. Questionnaires included data on demographics, socioeconomic status, lifestyle factors, physical activity, occupational history, circadian rhythm, mobile phone and pesticide use, past medical history, family history, medicine use, obstetrics and gynecology history, oral health, drugs and alcohol use, dietary habits and water use. Physical examinations included data on the pulse rate, blood pressure and anthropometric measurements (i.e. weight, height, waist circumference, hip circumference and wrist circumference). Biological samples included (fasting) blood, urine, hair and nail samples. Small amounts of blood and urine were used for biochemistry tests, and the rest plus nail and hair samples were stored in the PERSIAN biobank for future studies.^14,15^

 The PERSIAN cohort is due to follow participants for at least 15 years after enrollment. Individuals are annually contacted via phone calls. In cases of a death report, diagnosis of a major non-communicable disease, hospitalization, diagnostic/therapeutic care, or self-report of an event, a house/hospital visit will follow in order to obtain medical documents for further evaluation and determination of the event according to the tenth version of the international classification of diseases (ICD-10). Data collection in all phases is performed using a smart data server, which restricts possible common errors made during data entry.^14,15^

 The overall cohort methodology and rationale were published previously.^14,15^ Furthermore, the cohort data dictionary is available online (http://persiancohort.com/access/).^16^

## The PERSIAN Eye Cohort Study (PECS)

 The first phase of the PECS was launched as a specific ophthalmic branch of the PERSIAN Cohort at six of the existing cohort study sites in the January 2015 and was completed in September 2021 in the final center (Hoveizeh). The PECS aims to obtain ophthalmological information via an eye-specific history and an optometric examination. Fundus and slit-lamp photography were performed for the participants in the aforementioned six centers. During the optometric evaluation, individuals fulfilling certain criteria were referred for a comprehensive ophthalmological examination.^14,15^

**Figure 1:**
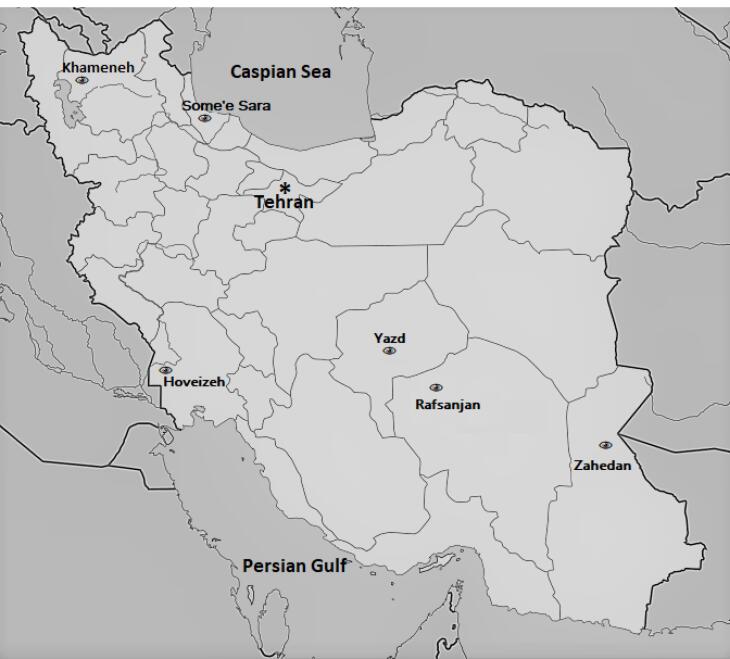
The PERSIAN Cohort Eye Study (PECS) Sites Throughout Iran, Inlcuding Khameneh, Some’e Sara, Hoveizeh, Yazd, Rafsanjan and Zahedan, Marked with an Eye Symbol. *Tehran: Capital of Iran

 The PECS sites are illustrated in Figure 1, while their details could be found in Table 1. These sites were selected according to ophthalmology facilities of the volunteering universities, as well as their geographical and ethnic distribution. They include Khameneh, Some’e Sara, Hoveizeh, Yazd, Rafsanjan and Zahedan, covering the major ethnicities of the Iranian people (including Persian, Turks, Arabs, Gilaks and Balouchs) as well as different climates (cold semi-arid in Khameneh; desert in Hoveizeh, Yazd, Rafsanjan and Zahedan; and the hot Mediterranean in Some’e Sara) of the country.^14,15,17^

**Table 1:** Characteristics of the Six Distinct Study Centers in the PERSIAN Eye Cohort Study (PECS), Iran, 2014–2020.

**City**	**Province**	**Major Ethnic Group(s)**	Climate*	**Biotope**	**Academic investigation Institution**
Khameneh	East Azerbaijan	Turk, Azari	BSk	Forest steppe	Tabriz UMS
Some’e Sara	Guilan	Gilaki	Csa	Forests, woodland	Guilan UMS
Hoveizeh	Khuzestan	Arab	BWh	Semidesert	Ahvaz Jundishapour UMS
Yazd	Yazd	Fars/Persian	BWh	Semidesert	Sadoughi Yazd UMS
Rafsanjan	Kerman	Fars/Persian	BWk	Semidesert	Rafsanjan UMS
Zahedan	Sistan and Balouchestan	Balouch	BWh	Desert lowlands	Zahedan UMS

PERSIAN, Prospective Epidemiological Research Studies in Iran; UMS, University of Medical Sciences. *Based on the Köppen Climate Classification ^13^; BSk, cold semi-arid climate; Csa, hot-summer Mediterranean climate; BWh, hot desert climate; BWk, cold desert climate.

 The PECS protocol was designed in a central committee located in Farabi Eye Hospital, Tehran, affiliated with Tehran University of Medical Sciences. This committee also defined the necessary settings and equipped the selected centers. Data collection was performed using a stepwise manner, starting with an optometry step which was performed by trained optometrists to ensure standard operation of instructions. All data collection was performed by an interviewer (an optometrist in the first step, and an ophthalmologist in the second step in cases who were referred for thorough ophthalmologic evaluation) who entered the data simultaneously via a web-based electronic platform (http://c.ddrc.ac.ir/PersianCohort). Therefore, the recorded data of participants could be monitored by the central committee at any time. Both steps were supervised by the central committee of the cohort centered in Farabi Eye Hospital, Tehran, Iran. Any patient who needed further evaluation was informed and referred to a tertiary center.

 The details of the optometry, ocular imaging and ophthalmology steps are explained in the following. A summary of the optometric step (questionnaire, examinations and ocular imaging), ophthalmologic step and equipment for each step could be found in Table 2. The data dictionary for the PECS is available online (http://persiancohort.com/access/).^18^

**Table 2:** Summary of Optometry Procedures Performed in the PERSIAN Eye Cohort Study

**Measurement**				**Reported as (unit)**	**Test**	**Equipment**
Optometry examinations	Visual acuity (VA)	Uncorrected (UCVA)		Illiterate E chart	—	Chart projector (CP-770, NIDEK, Japan), trial frame & trial lenses (TL-34P, SHIN-NIPPON, Japan)
		Best-corrected (BCVA) With present glasses (CCVA)				
	Refraction	Objective	Sphere	Diopter	—	Autorefractometer (AR1, NIDEK, Japan), Retinoscope (BETA^®^ 200, HEINE, Germany), trial frame & trial lenses (TL-34P, SHIN-NIPPON, Japan)
			Cylinder			
			Axis	Degree		
		Subjective	Sphere	Diopter	—	Trial frame & trial lenses (TL-34P, SHIN-NIPPON, Japan) & Snellen charts
			Cylinder			
			Axis	Degree		
	Lensometer	Glasses	Sphere	Diopter	—	Auto Lensometer (LM-500, NIDEK, Japan)
			Cylinder			
			Axis	Degree		
	RAPD			Positive/ negative	Swinging-flashlight test	Direct Ophthalmoscope (BETA^®^ 200, HEINE, Germany)
	Eye motility tests			Nystagmus/Exotropia/Esotropia/Hypotropia/Hypertropia	Cover-uncover test	
	IOP measurement (tonometry)			mmHg	—	Slit lamp (SL-3G, TOPCON, Japan) & Goldmann applanation tonometer (OPTILASA, Spain)
	Eye lid abnormality			Positive/ negative	Observation	-
	Corneal opacity			Positive/ negative	Observation	Slit lamp (SL-500, SHIN-NIPPON, Japan)
Ocular imaging	Slit lamp photography				—	Photo slit lamp (SL-500, SHIN-NIPPON, Japan) & camera (CANON, Japan)
	Fundus photography				—	Digital Retinal Camera (CR-2 AF, CANON, Japan)
Ophthalmological examinations	Eye lid/orbit abnormality			Positive/ negative	Observation	-
	RAPD			Positive/ negative	Swinging-flashlight test	Direct Ophthalmoscope (BETA^®^ 200, HEINE, Germany)
	Red reflex			Positive/ negative	—	Retinoscope (BETA^®^ 200 Streak, HEINE, Germany)
	Eye motility tests			Exotropia/esotropia/nystagmus	Cover-uncover test	
	Slit lamp biomicroscopy	Conjunctiva		Description and diagnosis	Observation	Slit lamp (SL-3G, TOPCON, Japan)
		Cornea				
		Lens				
		Vitreous				
		Anterior chamber angle		Open/occludable/closed	Van Herick test	
		IOP measurement (tonometry)		mmHg	—	Slit lamp (SL-3G, TOPCON, Japan) & Goldmann applanation tonometer (OPTILASA, Spain)
	Fundoscopy	Optic disc		Description and diagnosis	—	90 Diopter lens (Volk Digital Wide Field^®^, USA) & Slit lamp (SL-3G, TOPCON, Japan)
		Macula				
		Peripheral retina			—	20 Diopter lens (Volk Digital Wide Field^®^, USA) & indirect ophthalmoscope

## Optometry

 The main share of the PECS data is collected during the optometry step. The optometry step consists of three main parts: eye history questionnaire, optometry examinations, and imaging. This step took 20 minutes on the average to complete for each participant.

## Questionnaire

 The first step in data collection entails completing an interview questionnaire. In this step, the complete eye history of the participant was obtained. Information collected in this part included self-report of diabetes, wearing glasses/contact lenses, past year ophthalmic visits, amblyopia treatment, eye surgeries, dry eye symptoms, and family history of glaucoma, retinal detachment, keratoconus, and retinitis pigmentosa (RP).

## Examinations

###  Visual Acuity

 Visual acuity (VA) was measured for each eye using an Illiterate E chart and a chart projector placed in the standard distance based on the producers’ recommendation and was started with the right eye. First, VA was assessed without the usual distance correction of the participant (uncorrected VA, i.e. UCVA); therefore, the participants were asked to remove their spectacles or contact lenses.

 The test was considered complete when the individual was able to read at least half of the letters of the smallest line, and VA would be marked according to that line. If the participant was not capable of reading any of the letters at 1 meter, a finger counting test at different distances less than 1 meter would be performed. If the participant could count the fingers at a distance, the VA was recorded as finger counting at that distance. If still unable to count fingers at the closest distance, this was followed by the hand movement test at 30 centimeters. If still unable to detect the hand movement, this was followed by a light perception test. If the participant could detect the light, VA was recorded as light perception; otherwise, VA was recorded as NLP (no light perception). Then, the same process was repeated to measure corrected visual acuity (CCVA) with the patients wearing their habitual correction (eyeglasses or contact lenses).

## Refraction and Lensometer

 Objective and subjective refraction evaluations were performed for all participants. Objective refraction encompassed performing non-cycloplegic automated-refraction (autorefraction). The right eye was assessed first. Automated reports of sphere, cylinder and axis were recorded for each eye. Manual refraction was then performed by a trained optometrist using a manual retinoscope and loose lenses on the trial frame. During the objective refraction, poor red reflex and scissor motion signs were recorded, if present.

 For subjective refraction, a trial frame was adjusted on the individual’s face. Objective refraction estimates were used as the starting point, and alteration of sphere, cylinder and axis was performed guided by the patient’s answers to the Duochrome test and Cross-cylinder (0.25 D) until the best VA was achieved. Afterwards, the best-corrected visual acuity (BCVA) was measured using loose lenses on the trial frame.

 In cases of currently wearing near and/or far glasses, the glasses parameters were measured using an automated lensometer.

## Relative Afferent Pupillary Defect Test

 The presence of relative afferent pupillary defect (RAPD) was evaluated by the swinging light test, which is described in the following: first, the lighting of the room was dimmed. Then, the light source from a direct ophthalmoscope was shone from side to side into each eye, while the participant held their gaze on a distant object. The beam of light was held on each pupil for at least 3 seconds. Unilateral RAPD was defined as simultaneous dilation of both pupils when the light is shone into the affected eye.^19^

## Other Examinations

 The eye was examined for corneal opacities and eyelid lesions, such as ptosis, entropion, ectropion, etc. using a slit lamp. After instilling a drop of topical anesthesia into the inferior conjunctival sac and staining of the cornea with a dry strip of fluorescein, intraocular pressure (IOP) was measured using a Goldmann tonometer and IOP was reported in millimeters mercury (mm Hg). The right eye was assessed first. In cases with an estimated IOP of more than 20 mm Hg, the measurement would be repeated. The cover-uncover test was used to assess the presence of exotropia, esotropia, hypotropia and hypertropia. The presence of nystagmus was separately evaluated for each eye.

## Ocular Imaging

 The photography procedures started with an undilated slit lamp photography and proceeded with instilling two drops of topical tropicamide 1% in both eyes with a 5-minute interval between the drops. If the participants had a history of glaucoma or eye pain accompanied by evening headaches, an ophthalmologist’s permission for instillation was sought. After 20 minutes from the second instillation, the participant was led to the imaging section again, and underwent capturing 3 dilated slit-lamp photographs (slit-scan photo and retroillumination, needed for cataract grading, as well as 2 dilated fundus photographs (one macula centered and one optic nerve head centered). The instruction emphasized taking more photos in cases with an obvious pathology, focusing on the lesion.

## Slit-lamp Photography of Anterior Segment

 Slit lamp and anterior segment photography were performed using a Photo slit lamp (details mentioned in Table 2). Firstly, a diffused photo was taken using the diffuser option, capturing the eyelids, cornea, iris and lens. Once the pupils were dilated, two slit photos were captured from each eye with focus on the nucleus, from 45-degree angles. Afterwards, two retro-illumination photos were taken from 30-degree angles (right and left direction) from each eye. The World Health Organization (WHO) grading system was used for grading nuclear, cortical and posterior subcapsular cataract.^20^

## Fundus Photography

 The fundus photos were captured from the Early Treatment for Diabetic Retinopathy Study standard fundus fields 1 (which is focused on the optic disc) and 2 (focused on the macula),^21^ using a Digital Retinal Camera (details mentioned in Table 2).

## Ophthalmology Referral Criteria

 At the end of the optometry step, the optometrist discussed the findings of examinations with the participant. If the participant fulfilled any of the following criteria, they were referred for the next phase, which entails an appointment with an ophthalmologist: (1) Positive diabetes history, (2) Positive family history of glaucoma, (3) IOP > 20 mm Hg, (4) Positive RAPD, (5) BCVA < 8/10, (6) Documented/ suspicious strabismus, (7) Suspicious keratoconus, based on positive scissor motion sign, (8) Present eyelid abnormalities, (9) Moderate to severe dry eye symptoms, (10) Poor red reflex, (11) Any other suspicious findings.

 All participants required to undergo an ophthalmologic visit were booked an appointment by the secretary staff working at the cohort fields. The appointments were planned according to the ophthalmologists’ schedule in a way that no more than 30 patients were visited in one day.

## Ophthalmology Examinations

 Ophthalmic examinations were performed by an ophthalmologist in each center. At first, the eyelids, lacrimal system and extraocular muscles were assessed. Afterwards, slit lamp examinations were carried out using slit-lamp biomicroscopy which included evaluating the conjunctiva, cornea, anterior chamber angle (using Van Herick technique^22^), IOP, RAPD and red reflex.

 At this point in the examinations, the pupils were dilated using two sequential drops of tropicamide 1% with a 5-minute interval between instillations. The lens, vitreous, optic disc, macula and peripheral retina were examined after full mydriasis. A 90-diopter lens was used for posterior pole examination. Indirect ophthalmoscopy for assessing peripheral retina was also performed using a 20 diopter lens.

 At the end of the examination, a follow-up plan was designed according to the participant’s status: They were either followed up according to the study protocol without any further interventions, or were prescribed glasses, educated regarding lid hygiene/retinal detachment alarm signs. Those with serious concerns who needed further evaluation or surgical intervention were consulted and referred for further tests, consultation and surgery.

## Central Reading Center

 The PECS reading center team commenced working in the year 2020, and consisted of two panels of fundus photography and slit-lamp photography, led by board-certified subspecialists of cornea and retina as human reference standards and professional assessors who received thorough training and qualifications in classifying abnormalities. Each image is read by at least two assessors. In case of disagreement between the primary readers, the reference standard ophthalmologists act as a tiebreaker. All abnormal-labeled images will undergo an AI- (artificial intelligence) based process as well, in which different abnormalities will be distinguished by deep learning algorithms, trained by the AI committee at the beginning of the process. The AI committee is a collaborative committee with high-ranking members specializing in AI from the Institute for Research in Fundamental Sciences (IPM) of Tehran University and the central committee members.

## Data Monitoring

 As in the main PERSIAN Cohort, the Eye subcohort used a smart data server which limited common errors during data entry. This system also prevented entering out of range values for quantitative variables. Each center had its own focal point team, which acted as the liaison with the central team in supervising the whole process and addressing issues that arose. Furthermore, each center had a quality assurance (QA)/quality control (QC) team which was responsible for daily controlling of the data and weekly reports to the central team. Using the online data server, the central team was capable of checking the quality of online data entered by each center.

 Prior to beginning the work, the operators were informed that their voice and desktop performance will be recorded at times without their knowledge, which will be then appraised by the central team. The recordings were made using the FastSone Image Viewer software. In addition, routine unannounced inspections took place in each center by the PECS central team members to ensure adherence to protocols, optimal workflow, and equipment maintenance.

## Repeated Measurement

 The repeated measurement (RM) phase of the PECS was designed to start along with that of the main cohort, approximately five years after the enrollment. However, due to the effects of the COVID-19 pandemic, launching this phase is currently on hold.

 Nevertheless, the RM phase of the PECS will encompass repeating all the baseline steps, including eye questionnaire, optometry, ophthalmologic examinations and ocular imaging. A subset of 30% of the whole participants of baseline will be recruited, as well as those with referral indications.

## Ethical Approval

 The PERSIAN Eye Cohort Study proposal was approved by the ethics committees of the PERSIAN Cohort Study central team at DDRI, the MOHME, Tehran University of Medical Sciences (IR.TUMS.DDRI.REC.1396.1), as well as the six participating universities of medical sciences (Khameneh: tbzmed.rec.1393.205, Some’e Sara: IR.GUMS.REC.1394.226, Hoveizeh: IR.AJUMS.REC.1396.149, Yazd: IR.SSU.REC.1397.135, Rafsanjan: IRUMS.REC.1394.254, Zahedan: IR.ZAUMS.REC.1393.96451).

 All cohort participants were required to complete a written informed consent form prior to entering the Eye sub-cohort study. This was in addition to the primary consent provided at the enrollment phase of the main cohort. It was explained to each individual that participating in the eye sub-cohort is not obligatory and they could leave the study at any time.

## Demographic Characteristics

**Figure 2:**
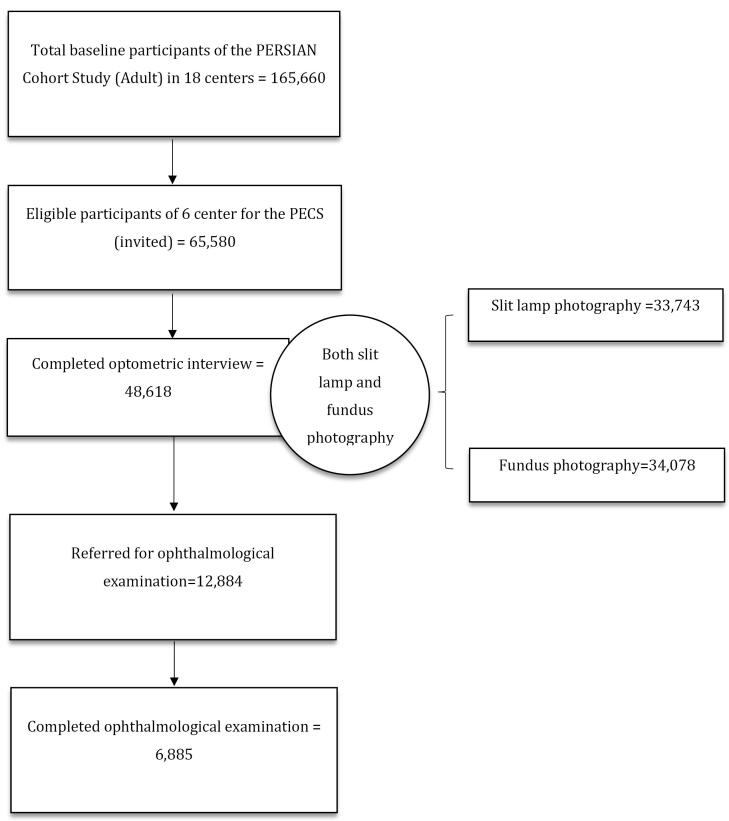
Flow Chart of the First Phase of the PERSIAN Eye Cohort Study (PECS), 2014–2021

 The first phase of PECS was launched in 2015, and completed in September 2021. The flow chart of the PECS first phase is depicted in Figure 2. The baseline demographics of participants who attended each step until now are represented in Table 3.

**Table 3:** Comparison of the Baseline Characteristics of the PERSIAN Cohort Participants of the Six Centers, Participants who Entered the PECS (optometry), and those who Completed Both Optometry and Ophthalmological Examination (2014–2021).

**Characteristics**		**Eligible participants of the 6 centers (n=65580)**	**Optometry (n=48618)**	**Optometry and ophthalmological examination (n=6885)**
Age at baseline (year), (mean ± SD)		49.52 (9.31)	49.91 (9.19)	54.76 (8.76)
Age groups (y), No. (%)	35-44	23 184 (35.35)	16 037 (32.99)	1052 (15.28)
	45-54	21 644 (33.0)	16 526 (33.99)	2116 (30.73)
	55-64	16 173 (24.66)	12 731 (26.19)	2708 (39.33)
	≥ 65	4579 (6.98)	3324 (6.84)	1009 (14.66)
Geographical location, No. (%)	Some’e Sara	10 520 (16.04)	7650 (15.73)	2315 (33.62)
	Khameneh	15 006 (22.88)	12 146 (24.98)	1816 (26.38)
	Zahedan	10 076 (15.36)	10 075 (20.72)	759 (11.02)
	Yazd	9978 (15.22)	7228 (14.87)	1177 (17.1)
	Rafsanjan	9991 (15.23)	8688 (17.87)	812 (11.79)
	Hoveizeh	10009 (15.26)	2831 (5.82)	6 (0.09)
Male sex, No. (%)		29 250 (44.6)	21636 (44.5)	2980 (43.28)
BMI, (mean ± SD)		27.55 (4.95)	27.54 (4.94)	27.43 (4.86)
Marital status (Ever married, No. (%)		64 364 (98.15)	47 861 (98.44)	6811 (98.93)
Socioeconomic status, (number, %)	Low	21 171 (32.32)	14 020 (28.86)	2511 (36.49)
	Average	24 568 (37.51)	18 668 (38.42)	2739 (39.81)
	High	19 761 (30.17)	15 897 (32.72)	1631 (23.7)
	Missing	80	33	4
Education, No. (%)	Illiterate (0 years)	12 527 (19.1)	7806 (16.06)	1588 (23.06)
	Primary education ( < 7 years)	25 809 (39.35)	19 405 (39.91)	3052 (44.33)
	Secondary education (8-12 years)	20 058 (30.59)	15 863 (32.63)	1769 (25.69)
	Tertiary education ( > 12 years)	7186 (10.96)	5544 (11.4)	476 (6.91)
Smoking status, No. (%)	Ever	14 422 (21.99)	10 405 (21.47)	1521 (22.12)
	Missing	350	145	9

 Out of 65 580 eligible participants of the six centers of the PERSIAN Cohort, 48 618 individuals aged 35-70 have been enrolled in the PECS (response rate: 74.13%) until June 2021. Slit lamp and fundus photography were performed for 28 702 (59.03%) and 27,437 (56.43%) individuals, respectively. The number of participants who were referred for an ophthalmology visit was 12 884, of whom 6885 completed the ophthalmology visit (response rate: 53.44%).

 Compared to the entire PERSIAN participants of the elected six centers, participants entering the PECS (i.e. those who completed the optometry step) are more likely to be older and have higher socioeconomic and education status. They are also more likely to have been married in their life at some point, and have a lower prevalence of ever smoking cigarette. Gender distribution and body mass index (BMI) are similar in both groups (Table 3).

## Discussion

 The PECS was designed to study the distribution of common ophthalmic conditions in a population of Iranian adults. Other aims of the study were to investigate the association of ocular diseases with systemic risk factors, as well as providing a base for future genetic research using the comprehensive biobank of the PERSIAN Cohort.

 Major ophthalmological cohort studies have investigated different populations across the globe, and have significantly contributed to expanding our knowledge of the eye.^3,23-26^ In Iran, the Shahroud Eye Cohort Study reported high prevalence of glaucoma and refractive errors in the adult population of Shahroud.^27-29^ The Tehran Eye Study found cataract to be a major issue in Iran’s capital due to its high prevalence and impact on vision, as almost one-third of visual impairments were caused by cataract.^30,31^ Another study in Varamin (a county in the Tehran province) found that the major barrier to receiving treatment in cataract patients was lack of awareness about the treatability of their condition.^32^ The findings of these studies were recognized as major causes of visual impairment in Iran and addressed in national prevention programs and policies of the MOHME, as a way to facilitate the WHO Vision 2020 global initiative in the country.^33^ Moreover, the findings of these studies have highlighted the need for more comprehensive ophthalmological research on a national scale, with a focus on the main visual problems of the Iranian populations according to the local studies. This is why the PECS decided to target cataract, refractive errors, glaucoma and diabetic retinopathy, as its main scope.

 While many eye cohorts have targeted European (the Netherlands, Germany, the United Kingdom),^3,23,26^ Australian,^6,34^ American^35^ and Asian (Chinese, Malay)^25,36,37^ populations, no multi-central prospective eye study has ever been conducted among a large sample of Middle Eastern populations. The PECS is exceptional in its design across the MENA/EMR region as it is the sole prospective eye cohort in this area with access to non-ophthalmic data as well as a large unique biobank for restoring tissues, useful for future genetic-based studies. The national design of the PECS, on the other hand, provides a great opportunity for looking at visual problems of the Iranian adults from a country-wide point of view, aiding the policymakers in prioritizing the interventions required for combating eye disorders in different regions of the country.

 Another focus of the PECS is investigating the association of socioeconomic inequality with prevalence of certain eye disorders. The Shahroud cohort study found that the gap of visual impairment between the low and high socioeconomic groups in Shahroud could be mainly explained by poor access to eye care services in lower economic groups.^38^ Using the data of the PECS, we will be able to identify socioeconomic barriers in receiving eye care resources and recognize regions requiring the most necessary measures for this aim.

 Previous studies have shown that by using retinal imagery, researchers can predict the prospect of cardiovascular outcomes of an individual. For instance, retinal vessel caliber has been suggested as a potential marker of coronary heart disease.^39^ Risk of stroke was also predicted by retinal arteriolar-to-venular diameter in the Rotterdam Study.^40^ Since all participants of the PECS will be followed for at least 15 years, the association between baseline retinal markers and future outcomes of vascular origin would be vastly explored.

 In comparison to the PECS, the first phase of the Shahroud Eye Study was launched in 2009-2010, and included 5190 participants aged 40–64 years. This study was exclusively conducted in Shahroud city.^11^ When comparing the PECS and the Shahroud Eye Study, it is noteworthy that the population structure of both eye cohorts is stable in terms of immigration, and both have been designed for follow-up eye measurements in order to investigate the effect of aging on ocular parameters. However, the PECS includes a wider age range (35–70 years). The Shahroud Eye Study focuses on diagnosis and detailed data collection on the eye, while PECS mostly aims to conduct screening of eye diseases (i.e. optometry and imaging step). The more detailed ophthalmology step was only performed in cases who were referred to the ophthalmologist, due to having a pathology or suspicious signs in the optometry step of the PECS. The population sample of the PECS covers six cities of Iran, and has nearly eight times the participants of the Shahroud Eye Study. Furthermore, the PECS has access to comprehensive information on the participants’ non-ophthalmic data, such as nutrition, sleeping habits, mobile phone use, job history and detailed medical data, while much of this information is missing from the Shahroud Eye Study.^11^

 The PECS benefits from several strengths. It has a large and stable sample size with a low migration rate and satisfactory response rate (74.13%), which covers the major ethnicities of the Iranian population living in different geographical regions. This allows for obtaining various exposures as risk factors to ophthalmic diseases, including diet, environmental and socioeconomic factors. In addition, some eye predictors which are less investigated in the literature are present in the PECS, including the nutritional data, sleeping habits, mobile phone use and occupational exposures. Access to systemic diseases and medical history is also very inclusive in the PECS. Furthermore, with regards to the genetic background of eye diseases in the MENA region, the PECS is by far the first eye cohort in the region to include a wholesome biobank. Future studies derived from this dataset will be able to demonstrate the association between specific gene-environment interactions of ocular diseases in this area.

 Another strong point of the PECS pertains to its objective imaging. More than 53% of all the respondents completed both slit and fundus photography, which would provide details on anterior and posterior segments of the eye. Mydriatic and non-mydriatic photography of both slit and fundus were done, which would enable accurate grading of cataract and retinal disorders, such as ARMD.

 There are some limitations to the PECS. Given the fact that the design of the PERSIAN Cohort Study does not include a random sample of Iran’s general population, the PECS also could not be considered an accurate representation of the Iranian general population. On the other hand, the PECS was conducted in six out of 18 centers of the PERSIAN Cohort. Comparing the respondents of the PECS with all eligible participants of the six centers shows that the respondents come from higher socioeconomic and educational classes. This could result in a selection bias. While these disparities limit the generalizability of the PECS, they are not unique to this study and have been reported by many similar cohorts, as well.^3,23,24^ As these cohorts also suggest, the large sample size and various exposures would still allow the PECS findings on the associations of exposures and outcomes to be valid. Furthermore, when reporting the prevalence rates of certain eye disorders, this bias could be tackled by applying adjustments and standardization methods at the time of analyzing the data.

## Conclusion

 In conclusion, in summary, the PECS is a large epidemiological multi-central eye cohort that originated from the ongoing PERSIAN Cohort Study. It was launched in 2014 to investigate the distribution of ophthalmic disorders in different regions and ethnicities of Iran, and determine their associations with various exposures of ophthalmic and non-ophthalmic nature. The next phase of the PECS (RM) will begin as soon as possible, with regard to the COVID-19 regulations in Iran. The vast array of exposures obtained in this study will also allow researchers to launch and study innovative hypotheses on the association of genetics and eye diseases in Iran. National and international research collaborations are welcomed through the PERSIAN Cohort website at http://persiancohort.com.
